# A volunteer-supported walking programme to improve physical function in older people with restricted mobility (the POWER Study): a randomised controlled trial

**DOI:** 10.1186/s12877-024-04672-4

**Published:** 2024-01-15

**Authors:** Nina Grede, Ulrike Trampisch, Sabine Weissbach, Monika Heinzel-Gutenbrunner, Ellen Freiberger, Andreas Sönnichsen, Norbert Donner-Banzhoff

**Affiliations:** 1 Institute of General Practice/Family Medicine, Philipps-University of Marburg, Marburg, Germany; 2 Department of Human Medicine, Institute of General Practice and Family Medicine, Faculty of Health, Witten/Herdecke University, Witten, Germany; 3 Department of Geriatric Medicine, Marien Hospital Herne, Ruhr University Bochum, Bochum, Germany; 4 Department of General Practice, Ruhr-University Bochum, Medical Faculty, Bochum, Germany; 5 Institute for Biomedicine of Aging, Friedrich-Alexander University Erlangen-Nürnberg, Nuremberg, Germany; 6 Research Initiative Health for Austria, Wissenschaftliche Initiative Gesundheit Für Österreich, Vienna, Austria

**Keywords:** Aged, Aged, 80 and over, Exercise, Accidental Falls, Quality of Life, Functional Status, Community Support, Randomised Controlled Trial

## Abstract

**Background:**

Regular physical activity has multiple health benefits, especially in older people. Therefore, the World Health Organization recommends at least 2.5 h of moderate physical activity per week. The aim of the POWER Study was to investigate whether volunteer-assisted walking improves the physical performance and health of older people.

**Methods:**

We approached people aged 65 years and older with restricted mobility due to physical limitations and asked them to participate in this multicentre randomised controlled trial. The recruitment took place in nursing homes and the community setting. Participants randomly assigned to the intervention group were accompanied by volunteer companions for a 30–50 min walk up to three times a week for 6 months. Participants in the control group received two lectures that included health-related topics. The primary endpoint was physical function as measured with the Short Physical Performance Battery (SPPB) at baseline and 6 and 12 months. The secondary and safety endpoints were quality of life (EQ-5D-5L), fear of falling (Falls Efficacy Scale), cognitive executive function (the Clock Drawing Test), falls, hospitalisations and death.

**Results:**

The sample comprised 224 participants (79% female). We failed to show superiority of the intervention with regard to physical function (SPPB) or other health outcomes in the intention-to-treat analyses. However, additional exploratory analyses suggest benefits in those who undertook regular walks. The intervention appears to be safe regarding falls.

**Conclusions:**

Regular physical activity is essential to preserve function and to improve health and quality of life. Against the background of a smaller-than-planned sample size, resulting in low power, and the interference of the COVID-19 pandemic, we suggest that community based low-threshold interventions deserve further exploration.

**Trial registration:**

The trial was registered with the German Clinical Trials Register (www.germanctr.de), with number DRKS00015188 on 31/08/2018.

**Supplementary Information:**

The online version contains supplementary material available at 10.1186/s12877-024-04672-4.

## Background

As people age, the risk of having two or more chronic somatic diseases and metabolic conditions increases rapidly [1]. In particular, frailty is a major challenge associated with the rapidly growing older population [2]. In this population, falls are common events with serious consequences for those affected and society in general. In addition to physical injuries, such as fractures, falls may lead to psychological consequences, such as fear of falling, social withdrawal, mood disorders and reduced quality of life (QoL) [3]. Due to the increasing proportion of older people worldwide, the ability to function within society at an increasing age is gaining importance. Therefore, the World Health Organization (WHO) has called to investigate disabilities (e.g. impairments, activity limitations and participation restrictions) in this vulnerable population [4].

Regular physical activity is recommended to improve the prognosis of chronic diseases in older people. The WHO recommends at least 2.5 h of moderate physical activity per week [5]. In fact, 73% of German women and 67% of German men aged ≥ 65 years do not meet this recommendation [6]. The benefits of regular exercise to reduce physical dependence in older people have been known for a long time [7–9]. Physical activity has long been established as a cornerstone of health and well-being, with numerous studies underscoring its positive effects on various health outcomes. While the benefits of structured exercise interventions are well-documented, the focus of this study diverges to explore a unique avenue—the potential impact of volunteer-supported programs on the health and well-being of older adults. Acknowledging the health benefits of exercise, our study seeks to shift attention to an alternative approach. Our intervention, focussed on the involvement of volunteers, to enhance the health and overall well-being of older adults. Importantly, apart from physical effects, we expect our interventions to increase social participation and improve intergenerational relationships.

The primary objective of this study is to assess the effectiveness of regular volunteer-supported outdoor walking compared with a control condition, the provision of unrelated health information. Specifically, we will examine its impact on physical function, cognitive function, frailty, fear of falling, and quality of life (QoL). The intervention was aimed at people ≥ 65 years old, who were not able to move independently and sufficiently due to physical limitations. In the first study period, the participating study centres provided the logistics for volunteer support. The second study period served to explore the possibility of non-academic initiatives to implement the idea of volunteer-assisted walks. Our study was previously conceptualised and piloted exclusively with nursing home residents [10]. Partly due to the very good uptake, we have now extended the setting to include majority community dwelling persons.

## Methods

### Study design

This randomised, controlled interventional superiority trial was undertaken from October 2017 to December 2021. The protocol was registered on 31 August 2018 at the German Clinical Trials Register (www.germanctr.de), Deutsches Register Klinischer Studien, with the number DRKS00015188. The study design has been published previously [11].

The study was carried out at primary care departments of the University of Witten-Herdecke (North-Rhine Westphalia) and the University of Marburg (Hesse), Germany. It was approved by Ethical Review Committee at both sites (Marburg reference number 208/17; Witten-Herdecke reference number 71/2018). Written informed consent was obtained from all participants and volunteers.

### Setting

Research personnel at both study sites recruited a cohort of participants ≥ 65 years and followed them up for 12 months. In the Witten region, we collaborated with nursing homes. The study team identified and approached potential participants. In the Marburg region, the study team recruited participants from the community setting. We involved primary care general practitioners (GP) and home care nursing services in the recruiting process. Moreover, the study was covered by local newspapers to encourage potential participants. Information leaflets were distributed in shops and pharmacies. Due to difficulties recruiting participants in the community, we also approached nursing homes in the Marburg region to recruit participants. A total of 224 participants were included.

### Volunteers

We used different channels to recruit volunteers. On the one hand, we approached cooperating partner organisations (e.g. volunteer agencies), and on the other hand we placed advertisements in local newspapers, internet forums and bulletin boards (e.g. of universities and schools).

The minimum age for volunteers was 16 years, which is the minimum age for helpers in the federal volunteering service (Bundesfreiwilligendienst), and the possession of a mobile phone. Moreover, volunteers were required to speak German sufficiently well, to be fit enough to assist participants during the walks and to be available for at least 6 months. The number of participants assigned to one volunteer was based on each volunteer’s time and the physical condition of the participants.

The study staff trained and prepared each volunteer for a total of 6 h. The training included instructions on how to support older people (e.g. using aids such as walkers) and how to document the walks. We then assigned the volunteers to the participants. We considered preferences during assignments (e.g. support of a female volunteer and participants close to home). We instructed the volunteers to arrange the appointments with the participants themselves.

### Participants

Participants were eligible if they were ≥ 65 years old and lacked confidence to a walk on their own, which we assessed informally. They had to have reduced physical function defined as a Short Physical Performance Battery (SPPB) score of < 9 [12]. For the pre-selection, we informed the nursing staff of the nursing homes as well as the participating GPs and nursing services about the inclusion criteria.

We excluded participants if they:

- did not give informed consent;
- had cognitive impairment (a Mini-Mental State Examination [MMSE] score of < 18 at baseline) [13];
- had severely reduced physical function so that volunteer-supported walks were not safe (an SPPB score at baseline of ≤ 2 in nursing homes and ≤ 3 in the community setting);
- had excellent physical function so that benefit from the intervention was unlikely (an SPPB score of ≥ 10);
- were permanently bedridden
- could only be mobilised in a wheelchair;
- already had regular physical activity levels estimated to be at least equivalent to the intervention;
- had a life expectancy of < 6 months as estimated by personal physicians and/or nursing teams;
- had another foreseeable inability to take part in the intervention for 6 months;
- had known alcohol or drug addiction or a psychotic episode during the last 12 months;
- another person of the same household already participated in the study.

Research staff visited potential participants who had either expressed an interest in the study or been identified by an institution and then screened them for eligibility.

### Randomisation

**Fig. 1:**
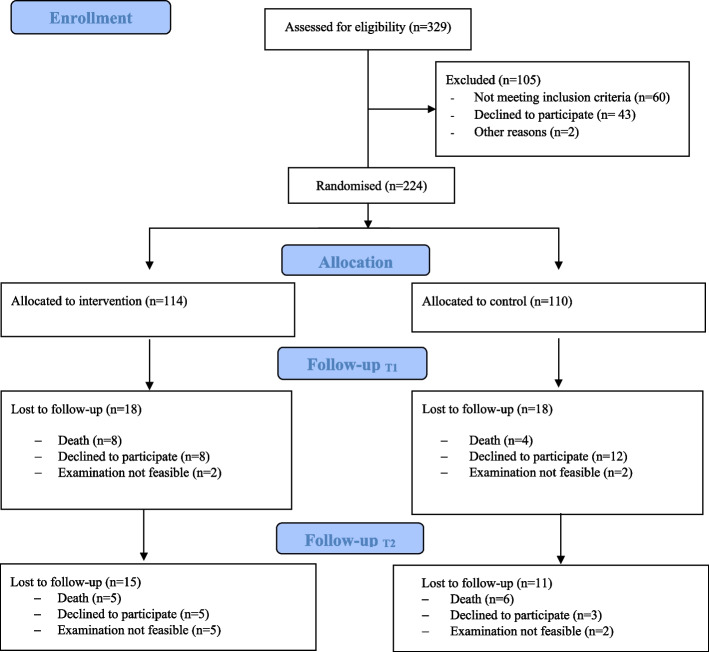
CONSORT Flow Diagram. *Participants to follow-up could still contribute to the ITT analysis, see Methods "Statistical analysis"

After completion of the baseline visit including checking of eligibility, we randomly assigned the participants to the control or experimental group according to the randomisation list, which was generated before the recruitment by the Clinical Trials Centre at the University of Marburg (Fig. 1). The randomisation stratified the two study centres with a blocking procedure, which created alternating blocks of 4 and 6 participants.

### Study visits

Research assistants performed the examinations and collected data at baseline (T0) and after 6 months (T1) and 12 months (T2) of the intervention. All visits took place at the participant’s private or nursing home. We collected sociodemographic data and characteristics like physical function and frailty at baseline. For details, see Table 1 and Supplementary Material S1.

**Table 1:** Baseline demographic and clinical characteristics for each group

	**Study arm**			
	**Control group *n* = 110**		**Intervention group *n* = 114**	
	**Median** **(IQR)**	**Min/Max**	**Median** **(IQR)**	**Min/Max**
**Age, years**	85.00[79.0–90.0]	65–98	84.00[80.0–90.0]	65–96
**BMI, kg/m**^**2**^	26.92[23.89–30.12]	16.8–44.1	24.96[22.83–28.73]	17.5–51.6
**Frailty scale**	4[3–6]	2–7	5[4–6]	2–7
**MMSE**	26[23–29]	18–30	27[23–29]	18–30
**Clock Drawing Test**	3[3, 4]	0–5	3[2–5]	1–5
**EQ-5D 5L VAS**	60[50–75]	9–100	60[50–80]	5–100
**FES-I**	30[21–39]	16–62	29[21–38]	16–56
**SPPB**	4[3–6]	2–9	4[3–5]	2–9
**Female sex, n (%)**	86 (78.2%)		92 (80.7%)	
**Speak German fluently, n (%)**	106 (96.4%)		114 (100%)	
**Family status, n (%)**				
- Widowed	78 (70.9%)		79 (69.3%)	
- Married	11(10%)		16 (14%)	
- Single	12 (10.9%)		8 (7%)	
- Divorced	9 (8.2%)		11 (9.6%)	
**Education level**				
- No educational qualification	3 (2.7%)		2 (1.8%)	
- Educational qualification (without vocational diploma)	52 (47.3%)		42 (36.8%)	
- Vocational school diploma	48 (43.6%)		59 (51.8%)	
- Academic degree	7 (6.4%)		11 (9.6%)	
**Need of nursing care**	***n* = 97**		***n* = 99**	
- Level 1	5 (5.2%)		7 (7.1%)	
- Level 2	49 (50.5%)		56 (56.6%)	
- Level 3	36 (37.1%)		29 (29.3%)	
- Level 4	7 (7.2%)		7 (7.1%)	

*BMI* Body mass index, *IQR* Interquartile range, *MMSE* Mini-Mental State Examination, *VAS* Visual Analogue Scale, *FES-I* Falls Efficacy Scale, *SPPB* Short Physical Performance Battery

### Intervention

After baseline assessments (T0) and randomisation, participants in the intervention group received the physical activity intervention for 6 months. They were visited by an assigned volunteer up to three times a week to go for a walk outside. The initial duration and speed of the walk were determined according to the participant’s physical ability. The aim was to gradually increase the duration of each walk up to 50 min to meet the WHO recommendation of 150 min per week [5]. The activity could take place indoors in case of bad weather under the supervision of the volunteer. It consisted of exercises for balance and strength based on a programme of the federal centre for health education [14]. This brochure provides simple, illustrated instructions for effective and safe indoor training (see Supplementary Material S4). The study intervention is described in more detail elsewhere [11].

Walking pairs of participants and volunteers received an activity diary to record the date, time, duration and type of each exercise episode (outdoors or indoors). Events relevant for the safety of the intervention, such as falls or injuries, were also documented in the diary by the walking pairs themselves. After each walk, the participant recorded their subjectively experienced physical strain on a visual analogue scale. We invited the participants in the control group to two lectures given by study staff. The lectures covered topics related to healthy ageing, such as diet or the interpretation of blood tests. We presented the topics in an easy-to-understand and entertaining manner. These lectures did not mention physical activity.

### Follow-up and extension study

After 6 months, the post-intervention examination took place for both study arms (T1). We followed the study participants in both groups for an additional 6 months until the final examination at 12 months (T2). During this second study period, the academic study centres did not organise or coordinate walks. However, we expected community services outside the coordinating academic departments to continue to provide support for regular walking as initiated. The evaluation of the extended period at T2 had two main objectives: first, to prolong the follow-up and to assess the long-term effects of regular walking (up to 12 months); second, to evaluate potential sustainability and dissemination of the study intervention after cessation of support from the academic study centres.

### Outcomes

The primary outcome of the study was physical function as measured by the SPPB. This assessment includes several observed activity tests measuring balance, gait speed and the ability to get up from a chair. SPPB scores are associated with disability in mobility and ADL [15–17], future hospitalisation [18], health improvement [19] and mortality [19–21]. The SPPB has good reliability and validity [16].

The secondary outcomes were quality of life (the EQ-5D-5L score) [22], fear of falling (the Falls Efficacy Scale [FES-I]) [23], physical activity (activity diary) and cognitive executive function (the Clock Drawing Test [CDT]) [24]. We defined falls requiring medical attention, any hospitalisation and death as adverse events. We obtained details on these events from the participants’ primary care physicians and/or from hospital discharge reports. See Supplementary Material S1 for an overview of the measurements at T0, T1 and T2. Some of these findings will be published separately.

### Blinding

Due to the type of intervention, neither participants nor volunteers could be blinded regarding the intervention. Because study staff communicated with participants at T0, T1 and T2 for at least 30 min, shielding them from information regarding the study arm was not a realistic option. However, randomisation took place after the baseline examination by an independent unit, thus ensuring allocation concealment.

To keep data collection at follow-up visits as unbiased and consistent as possible, we developed a standardised protocol. The study statistician (MHG) received datasets without labels regarding group allocation; thus, they were blinded to the allocation of the participants.

### Sample size

For our sample size calculation, we chose physical function as the primary outcome – that is, the SPPB score at T1. A change of 1 point has been shown to be of clinical relevance (e.g. predictive of future hospitalisation, health improvement and mortality) [25]. The standard deviation in comparable samples has ranged from 2.6 to 2.8 points [19, 24, 25]. We chose a conservative approach and assumed the higher standard deviation (2.8) for our calculation. In an analysis of covariance (including the baseline values of the primary endpoint), with R^2^ of 0.5 for the covariate and a power of 95% (1 – β), a sample size of 206 would be required to detect a difference of 1 point between the means of the two study arms.

In the pilot study we conducted [10], which we initiated solely in nursing homes, we found a 28% loss of participants during the 6-month intervention period mainly due to death and hospitalisation. Because we did not know what loss could be expected in the community setting, we chose a conservative approach with an estimated expected loss of 40% of participants during the first intervention period, mainly due to death and hospitalisation. Hence, a total sample of 345 people would be required to obtain a sufficient power of 95% for our primary endpoint. The Marburg Study Centre aimed to enrol 230 people from the community and the Witten study centre aimed to enrol 115 people from nursing homes.

### Statistical analysis

We used SPSS Statistics Version 27 (IBM Corp., Armonk, NY, USA) for all analyses. Metric outcomes are reported as the mean and standard deviation if they were normally distributed, or as median and interquartile range if they were not normally distributed.

For the primary efficacy analysis of the intention to treat (ITT) population, we hypothesised a higher SPPB score at T1 in the intervention group compared with the control group. To evaluate the difference in the primary outcome (the SPPB score) between the treatment groups, we applied linear regression with a robust estimator of the covariance matrix in the framework of generalised linear models with the predictors treatment and the baseline SPPB score [26]. Thus, the result is controlled for by the baseline SPPB score.

We evaluated the secondary outcomes – SPPB at T2, QoL (the EQ-5D-5L score) at T1 and T2, cognitive function (the CDT score) at T1 and T2, fear of falling (the FES-I score) at T1 and T2 and physical activity at T1 and T2 with Student’s t-tests or the Mann–Whitney U-test. We determined whether the data met the assumptions for the parametric models, namely a normal distribution, by inspecting the Q–Q plots.

We postulate two reasons for missing values of outcome variables. Nursing home or hospital admission as well as death can be regarded as ‘informative’ because they are potentially related to the intervention. In other words, the study intervention is intended to reduce hospital admissions and deaths. In our view, however, moving out of the area should be regarded as ‘non-informative’, because it is not related to the intervention. We ascertained the reason for loss to follow-up. For the ITT analysis, we replaced missing values according to the reason they were missing. For ‘informative’ missing values, we substituted the worst possible value of the respective variable. For ‘non-informative’ missing values in the primary outcome, we proceeded with the multiple imputation procedure of SPSS Statistics Version 27. The approach resulted in 10 complete datasets. The imputation was based on the non-missing baseline variables sex, age, frailty, the FES-I score and the SPPB score. We used the ‘METHOD’ keyword ‘AUTO’, which is default and specifies the imputation method.

We analysed the primary outcome in the per protocol (PP) dataset by performing a sensitivity analysis. We included participants attending the T1 visit and who, if in the intervention arm, had completed at least 25% of their scheduled walks (equal to 20 walks). We used the χ^2^ test to compare deaths between the groups and Poisson regression to compare falls between the groups. Based on imputation and replacements, patients lost to follow-up could contribute to our primary outcome analysis. The remaining outcome evaluations were dependent on compliance with study procedures. Thus, the numbers differ according to the availability of data (see Tables 2 and 3).

**Table 2:** Primary and secondary outcomes in the intention-to-treat (ITT) population

	**Outcome**	**Study arm**				
		**Control group** **T1: *n* = 97, T2: *n* = 82**		**Intervention group** **T1: *n* = 105, T2: *n* = 85**		
**Time**		**Median**	**IQR**	**Median**	**IQR**	***p***
**T1**	**SPPB**	3.00	[2.00–5.00]	4.00	[3.00–5.00]	0.308^a^
	**Frailty scale**	5.00	[4.00–6.00]	5.00	[3.00–6.00]	0.281^b^
	**Clock test**	3.00	[2.00–4.00]	3.00	[2.00–4.00]	0.799 ^b^
	**EQ-5D 5L VAS**	55.00	[45.00–75.00]	50.00	[45.00–70.00]	0.355 ^b^
	**FES-I**	28.00	[20.00–38.00]	27.00	[21.00–37.00]	0.805 ^b^
**T2**	**SPPB**	3.00	[2.00–5.00]	4.00	[2.00–5.00]	0.225 ^a^
	**Frailty scale**	6.00	[4.00–6.00]	5.00	[4.00–6.00]	0.912 ^b^
	**Clock drawing test**	3.00	[2.00–4.00]	3.00	[2.00–4.00]	0.661 ^b^
	**EQ5D 5L VAS**	50.00	[47.50–72.50]	54.00	[50.00–75.00]	0.425 ^b^
	**FES-I**	27.00	[20.00–38.00]	26.00	[20.00–35.00]	0.964 ^b^

*IQR* Interquartile range, *FES-I* Falls Efficacy Scale, *SPPB* Short Physical Performance Battery, *VAS* Visual Analogue Scale

Informative missing values were replaced by the worst outcome possible

^a^Generalised linear model, controlled for the baseline SPPB score

^b^Mann–Whitney U test if the data were not normally distributed

**Table 3:** Safety analyses

		**Study arm**				
		**Control group** **T1: *n* = 94/110, T2: *n* = 83/97**		**Intervention group** **T1: *n* = 100/114, T2: *n* = 84/105**		
		**Count**	**%**	**Count**	**%**	***p***
**T1**	**Number of falls**					
	**0**	66	60.0	65	57.0	**0.668**^**a**^
	**1**	11	10.0	19	16.7	
	**2**	9	8.2	4	3.5	
	**3**	2	1.8	3	2.6	
	**4**	2	1.8	1	.9	
	**Death**	4	3.6	8	7	**0.375**^**b**^
**T2**	**Number of falls**					
	**0**	60	54.5	62	54.4	**0.942**^**a**^
	**1**	12	10.9	10	8.8	
	**2**	3	2.7	3	2.6	
	**3**	2	1.8	3	2.6	
	**4**	2	1.8	2	1.8	
	**Death**	4	3.6	4	3.5	**1**^**b**^

^a^Poisson regression

^b^χ^2^test

## Results

### Participants

The participant flow is summarised in Fig. 1. The study sample comprised 224 participants. Because of difficulties recruiting participants in the community (planned sample size for Marburg n = 230, achieved *n* = 106), we failed to achieve the overall planned sample size of 345 participants. We recruited 118 participants in Witten, all of whom were nursing home residents. Most participants in the Marburg region lived in the community (*n* = 76 [72%]); the remaining 30 (28%) lived in nursing homes. Overall, 79% of the sample was female. A total of 196 (87.5%) participants had an officially acknowledged need of nursing care (in German, Pflegegrad). Of these participants, 65 (31.2%) had at least level 3 (of 5 [= worst]). We randomised 110 participants into the control group and 114 into the intervention group. Table 1 shows their characteristics at baseline.

### Compliance with the trial protocol

The overall number of walks by each participant in the intervention group ranged from 0 to 101 with a mean ± standard deviation (SD) of 17.7 ± 19. Assuming walks three times a week over a period of 6 months, this would result in 78 walks. We included only the participants in the intervention group with at least 20 walks, 40 participants, in the PP population. Community participants completed 20 walks more often (37%) than nursing home participants (20.8%). The overall number of indoor training, in case of bad weather, by each participant in the intervention group ranged from 0 to 17 with a mean ± standard deviation (SD) of 3.4 ± 2.5.

### Efficacy of the intervention

The effects of the intervention on the primary and the secondary outcomes evaluated in the ITT population are presented in Table 2. There were no significant differences in our primary outcome, the SPPB score, frailty, QoL, cognitive function or fear of falling between the study arms at T1 or T2.

Given the interference of the COVID-19 pandemic with the study intervention and visits, we conducted a PP analysis for our primary outcome at T1 (*n* = 40). In the PP population, the SPPB scores of participants who actively took part in the intervention were higher than those of the controls (mean ± SD: 4.82 ± 2.46 vs. 3.87 ± 2.56, *p* = 0.01). As an additional exploratory analysis, we applied a regression model to the intervention group only, with the SPPB score at T1 as the dependent variable. As predictors we chose the baseline SPPB score and the ‘number of walks completed’. The latter had a significant influence on the outcome. We repeated the same analysis with ‘time spent walking’ as the independent variable (for details, see Supplementary Material S3).

### Safety

During the study, there were no significant differences in falls and death between the groups at T1 or T2 (Table 3). None of the recorded falls or hospitalisations were associated with volunteer-assisted walks. The number of days spent in hospital between baseline and T1 was 236 in the intervention arm and 267 in the control arm (analysis of variance [ANOVA] *p* = 0.771), and between T1 and T2 it was 109 in the intervention arm and 259 in the control arm (ANOVA *p* = 0.151). We obtained similar results for the number of hospitalisations (see Supplementary Material S2).

### Post hoc power analysis

Because of the impact of the COVID-19 pandemic on the study, there are large discrepancies between the PP and ITT populations. Would our study have been more successful if the participants had adhered to the study protocol? Assuming an effect size as shown in the PP analysis and a sample size of 202, the conditional power calculation method suggests 77% power to detect an R^2^ of 0.019 attributed to one independent variable using an F-test with a significance (alpha) level of 0.05. The variables tested are adjusted for an additional covariate, which has a combined R^2^ of 0.454 by itself.

## Discussion

We could not show that regular, volunteer-assisted walks for old people improve physical or cognitive function, frailty, fear of falling or QoL. The intervention, aimed at individuals ≥ 65 years with reduced physical function and lacking confidence and/or external support, appears to be safe regarding falls. Although ITT comparisons were negative, exploratory analysis suggested a positive effect of walking on health outcomes.

### Difficult recruitment

While recruitment in nursing homes proceeded as planned, finding participants in the community proved to be difficult. Our eligibility criteria apparently applied to only to a small section of the population aged ≥ 65 years old in the community. We wanted to reach individuals restricted in their physical function who lacked opportunities and support for regular physical activity. We excluded bedridden people or those in whom mobilisation seemed unlikely to succeed. Potential participants were pleased to meet volunteers; the walking part, however, deterred many [27]. GP practices and home care nursing services were often too busy to approach patients systematically regarding study participation. Therefore, we decided to use additional channels to reach our target population, such as local newspapers, flyers in shops, etc. Although these measures were successful to a certain degree, we did not reach our recruitment target within the planned time period. Cooperation and recruitment were far more straightforward in nursing homes. Management was usually happy to offer residents additional activities. Hence, they showed considerable commitment towards the study.

### Negative result

The contact restrictions due to the COVID-19 pandemic had an additional impact on achieving the study objectives. Walks were cancelled, which led to some dropouts, but also to relevant delays in study visits to evaluate outcomes. The second study period suffered the most from the COVID-19 pandemic. Its objective was to explore whether actors in the community would continue to offer exercise support to the older population. Restrictions related to the COVID-19 pandemic made this largely impossible.

### Promoting well-being in older people through volunteer support

Various consortia address healthy aging, such as the WHO Clinical Consortium on Healthy Ageing [28]. The evidence supporting the positive impact of a physically active lifestyle on the health of older adults is substantial; however, only a limited percentage of the elderly population adheres to the recommended levels of physical activity.

Whithall and colleagues undertook a qualitative analysis of the best approaches and synthesised evidence from end-user representatives and stakeholders to refine one of these approaches, an intervention to promote active ageing through peer volunteering [29]. They state that participants engaged primarily for social reasons, facing barriers like lack of companionship, low confidence, weather concerns, and established group dynamics. Volunteers emphasized the need for meaningful engagement and social interaction. The study supports peer-volunteering for active aging, emphasizing effective recruitment and overcoming barriers like lack of motivation and security concerns. This study is based on the findings of the study by Stathi and colleagues [30]. ACE (Active, Connected, Engaged) was a feasible and well-accepted intervention using peer-volunteering support to promote active aging in socially disengaged older adults. The study, involving 54 participants, demonstrated that ACE increased out-of-house activities, improved physical function, and enhanced well-being and vitality. Participants in the intervention reported increased confidence, knowledge of local initiatives, and perceived social support. The findings emphasize ACE's potential to help socially disengaged older individuals get outdoors, boost confidence, and engage more with their community.

The interest in volunteer support for the walks in our study was highly positive. We received strong interest and support from the public, stakeholders, and other interest groups. The sub-study conducted by Weissbach et al. successfully demonstrated this in a mixed-method approach [27]. Both participants and volunteer companions reported, in semi-structured guide-based interviews (nursing home residents), two focus group interviews (volunteers), and a cross-sectional questionnaire survey (volunteers), not only physical improvements but also highlighted the positive impact of social interaction associated with the walks. The study findings indicate that volunteer support for mobility-impaired nursing home residents has a positive impact on the quality of life for both groups. The simple intervention received predominantly positive evaluations, even though no new insights into physical activity were gained. Future programs should be tailored to the individual needs of older adults to enhance their quality of life and mobility. A suitable environment in the nursing home and training for volunteers are crucial for the success of such initiatives.

### Strengths and limitations

Despite our failure to achieve our recruitment goals, our study had some strengths. We were able to recruit, motivate and instruct a sufficient number of volunteers to support the study participants in their walking. Most participants and volunteers enjoyed the experience.

Our main outcome was a battery of physical function tests. A study with sufficient power to investigate outcomes such as QoL or frailty would be desirable. Because of the nature of the intervention, the participants could not be blinded. Due to logistical constraints, we also could not blind the study personnel. Given the clear preferences some (potential) participants had, (potential) allocation to the ‘wrong’ study arm made motivation to contribute to the project sometimes difficult to maintain.

We also found that acute health problems, bad weather conditions, volunteers moving or lacking time proved to be obstacles. When establishing a permanent service as evaluated in this study, planners should keep in mind that it requires high flexibility. Community organisations such as public health departments or municipal volunteer agencies were highly interested in supporting the idea underlying the study. However, the COVID-19 pandemic prevented us from exploring this promising aspect further.

## Conclusions

To our knowledge, this is the first randomised controlled trial evaluating a low-threshold intervention such as volunteer-assisted outdoor walking to improve physical function in older people. Against the background of a smaller-than-planned sample size resulting in low power, and the interference of the COVID-19 pandemic, we suggest that the idea of community-based low-threshold interventions of this kind should be explored in future studies.

### Supplementary Information

**Additional file 1.**

## Data Availability

The results of the data analysis are available from the corresponding author upon reasonable request.

## Abbreviations

ADL: Activities of daily living
CDT: Clock Drawing Test
COVID-19: Coronavirus disease 2019
DRKS: Deutsches Register Klinischer Studien (German Clinical Trials Register)
FES-I: Falls Efficacy Scale
GP: General practitioner
ITT: Intention-to-treat
MMSE: Mini-Mental State Examination
PP: Per protocol
QoL: Quality of life
SPPB: Short Physical Performance Battery
WHO: World Health Organization
