# Supplementary material for: A volunteer-supported walking programme to improve physical function in older people with restricted mobility (the POWER Study): a randomised controlled trial
Source: BMC Geriatr. 2024 Jan 15;24:60. doi: 10.1186/s12877-024-04672-4 (PMC10789062; doi:10.1186/s12877-024-04672-4)
Supplement: Supplementary file 1 — Additional file 1. [file 12877_2024_4672_MOESM1_ESM.docx]

# The POWER Study: Supplementary Material

# S1 Measures at baseline and follow-up assessments

| **Outcome measure** | **Operationalisation**  **(type of assessment)** | **Time of assessment** | | |
| --- | --- | --- | --- | --- |
|  |  | **T0** | **T1** | **T2** |
| **Participant** | | | | |
| Sociodemographic data | Age, sex, level of care, family status, ethnicity | X |  |  |
| Physical function | Short Physical Performance Battery [18] | X | X | X |
| Quality of life | EQ-5D-5L [28] | X | X | X |
| Fear of Falling | Falls Efficacy Scale [29] | X | X | X |
| Frailty | Clinical Frailty Scale [34] | X | X | X |
| Cognitive changes | Clock Drawing Test [30] | X | X | X |
| Cognitive impairment | Mini-Mental State Examination [19] | X |  |  |
| Walk assessment (activity diary) | Number and duration of walks, assistance, type of assistance |  | X | X |
| Falls | Type and place of treatment |  | X | X |
| Treated falls | Date, ICD code, falls |  | X | X |
| Hospitalisation | Date of hospital admission and discharge, duration of hospitalisation, reason for hospitalisation (ICD-10 code) |  | X | X |
| Death | Date of death, reason for death (ICD-10 code) |  | X | X |

ICD, International Classification of Disease

# S2 Additional safety analyses

| Tabulation of study arm × number of hospitalisations from baseline to T1 | | | | | | | | |
| --- | --- | --- | --- | --- | --- | --- | --- | --- |
|  | | | Count (%) | | | | | Total |
|  |  |  | 0 | 1 | 2 | 3 | 4 |  |
| Study arm | Control group | Count | 98 | 5 | 3 | 1 | 3 | 110 |
|  |  | Row % | 89.1 | 4.5 | 2.7 | 0.9 | 2.7 | 100.0 |
|  | Intervention group | Count | 100 | 8 | 5 | 0 | 1 | 114 |
|  |  | Row % | 87.7 | 7.0 | 4.4 | 0.0 | 0.9 | 100.0 |
| Total | | Count | 198 | 13 | 8 | 1 | 4 | 224 |
|  |  | Row % | 88.4 | 5.8 | 3.6 | 0.4 | 1.8 | 100.0 |

In the control group, 11% of the participants were hospitalised at least once. In the intervention group, 12% of the participants were hospitalised at least once. Hence, there was an average of 0.24 (standard deviation = 0.79) hospitalisations in the control group and 0.19 (standard deviation = 0.59) hospitalisation in the intervention group.

| Tabulation of study arm × number of hospitalisations from T1 to T2 | | | | | | | | |
| --- | --- | --- | --- | --- | --- | --- | --- | --- |
|  | | | Count (%) | | | | | Total |
|  |  |  | 0 | 1 | 2 | 3 | 4 |  |
| Study arm | Control group | Count | 96 | 8 | 3 | 1 | 2 | 110 |
|  |  | Row % | 87.3 | 7.3 | 2.7 | 0.9 | 1.8 | 100.0 |
|  | Intervention group | Count | 102 | 8 | 3 | 1 | 0 | 114 |
|  |  | Row % | 89.5 | 7.0 | 2.6 | 0.9 | 0.0 | 100.0 |
| Total | | Count | 198 | 16 | 6 | 2 | 2 | 224 |
|  |  | Row % | 88.4 | 7.1 | 2.7 | 0.9 | 0.9 | 100.0 |

There was an average of 0.23 (standard deviation = 0.71) hospitalisations in the control group and 0.15 (standard deviation = 0.48) hospitalisations in the intervention group.

# S3 Regression analyses

Evaluation of the total number of walks and total walking time in the intervention group

The distribution characteristics of total number of walks and total walking time in the intervention group are tabulated below.

|  | Number | | Mean | Standard deviation | Median | Minimum | Maximum |  |
| --- | --- | --- | --- | --- | --- | --- | --- | --- |
|  | Valid | Missing |  |  |  |  |  | IQR |
| Total number of walks | 111 | 3 | 17.69 | 18.97 | 12.00 | 0.00 | 101.00 | 3; 26 |
| Total walking time [hours] | 90 | 24 | 16.84 | 13.71 | 13.50 | 0.33 | 72.95 | 5.8; 23.2 |
|  |  |  |  |  |  |  |  |  |

IQR, interquartile range

A linear regression with the dependent variable Short Physical Performance Battery (SPBB) score at T1 and the potential influence factors ‘total number of walks’ and ‘total walking time [h]’ and controlled for by the SPBB score at baseline resulted in R^2^ = 0.51

The results of linear regression are tabulated below, showing a significant association between ‘total walking time [hours]’ and the SPBB score at T1.

|  | | | | | | |
| --- | --- | --- | --- | --- | --- | --- |
| Model | | Unstandardised coefficients | | Standardised coefficient | t | p |
|  |  | B | Standard error | Beta |  |  |
| 1 | (Constant) | 0.053 | 0.511 |  | 0.104 | 0.918 |
|  | BL_SPPB | 0.757 | 0.117 | 0.535 | 60.458 | <0.001 |
|  | Total walking Time [hours] | 0.076 | 0.024 | 0.438 | 30.221 | 0.002 |
|  | Number of walks | -0.022 | 0.017 | -0.174 | -10.318 | 0.191 |

# S4 Broschure link

https://shop.bzga.de/pdf/60582359.pdf
